# Barrier-Oriented FWGM-Based Fuzzy-FMEA for Risk Assessment and Safety-Barrier Prioritization in Solvent-Based Electrospinning Processes

**DOI:** 10.3390/ma19122673

**Published:** 2026-06-22

**Authors:** Jong Gu Kim, Byong Chol Bai

**Affiliations:** 1 Department of Fire and Disaster Prevention and Safety, Sun Moon University, Asan 31460, Republic of Korea; jonggukim@sunmoon.ac.kr; 2 Department of Chemical Engineering, Daejin University, Pocheon 11159, Republic of Korea

**Keywords:** electrospinning, fuzzy-FMEA, process safety, safety barriers, nanofiber manufacturing

## Abstract

This study proposes a barrier-oriented application of conventional failure mode and effects analysis (FMEA) and fuzzy weighted geometric mean (FWGM)-based fuzzy-FMEA for laboratory-scale solvent-based electrospinning. The process was decomposed into 14 sequential steps, and one representative failure mode was defined for each step. Severity, occurrence, and detection were rated by a five-member expert panel, and hazard-type-specific weights were assigned to chemical-dominant, electrical-dominant, fire/static-dominant, and combined-dominant hazards. Conventional FMEA identified material review/approval, equipment setup, pre-start inspection, and response to abnormalities as the highest-risk steps (RPN = 60). FWGM-based fuzzy-FMEA re-ranked tied RPN groups and identified response to abnormalities and equipment setup as the joint highest-FRPN failure modes (FRPN = 79.35), followed by pre-start inspection (77.39) and material review/approval (75.89). Barrier-oriented interpretation revealed four dominant mechanisms: upstream information-based hazards, direct high-voltage access, pre-start combined hazards, and intervention under abnormal or residual-energy states. Scenario-based post-control analysis showed that grounded enclosures, interlocks, de-energize-discharge-verify procedures, pre-start checklists, and bonding/grounding measures reduced FRPN by 25.88–43.79% for prioritized failure modes. The proposed framework supports SOP development, equipment improvement, training prioritization, and laboratory risk-assessment documentation for solvent-based nanofiber manufacturing.

## 1. Introduction

Electrospinning is a representative process for producing continuous nanofibers by applying an electric field to a polymer solution or melt. Because it can generate high specific surface area and porous structures, it has been widely used in separators, filtration media, energy materials, and biomaterials [1,2,3,4]. However, process performance and operational stability are highly sensitive to operating variables such as applied voltage, flow rate, tip-to-collector distance, and solvent volatility, and even small deviations in laboratory-scale systems can lead not only to product-quality problems but also to safety issues [5,6,7,8].

In particular, solvent-based electrospinning is a multi-hazard process in which the toxicity, volatility, and flammability of organic solvents coexist with the electrical hazards of high-voltage equipment. During operation, inhalation and dermal exposure to chemicals, accumulation of flammable vapors, electrostatic ignition, electric shock, arcing, and secondary accidents caused by residual charge may overlap. In addition, residual charge and stored energy can remain inside the system even after power shutdown; therefore, simple power-off cannot be regarded as sufficient to ensure high-voltage safety [9,10,11].

For these reasons, risk assessment for electrospinning requires more than a simple list of hazards; it needs a structured framework that can proactively identify failure modes at each process step and examine their causes, consequences, and existing controls together. In laboratory-scale electrospinning, sequential work steps are clearly distinguished—including solution preparation, equipment setup, pre-start inspection, voltage application, response to abnormalities, shutdown, and maintenance—and the level of operator intervention and energy state can vary substantially from one step to another [11,12].

Previous studies on electrospinning have largely focused on identifying relationships between process variables and fiber diameter, bead formation, pore structure, and functionality, with the aim of optimizing material performance [13,14,15,16]. Although some studies and laboratory safety guidance suggest basic precautions such as ventilation, grounding, shielding, and the use of personal protective equipment, these discussions have generally remained at the level of individual precautions and have not structured step-specific failure modes together with existing safety barriers [9,10,11].

To address this gap, risk assessment can employ various analytical methods, including Fault Tree Analysis, Event Tree Analysis, and Layer of Protection Analysis [17,18,19]. However, laboratory and development-stage processes are characterized by variable procedures and operating conditions and by limited accident or failure data. Under such conditions, an approach is needed that can rapidly identify hazards and set improvement priorities before detailed quantitative analysis. Failure mode and effects analysis (FMEA) is well suited to this purpose because it can systematically structure failure modes by process step and prioritize them using severity (S), occurrence (O), and detection (D), even when large-scale statistical failure data are unavailable [20,21].

However, conventional FMEA alone is insufficient to capture the composite hazards of electrospinning. Failure modes such as direct access to high voltage, residual charge, solvent exposure, and ignition hazards involve considerable ambiguity in expert judgment because their hazard boundaries are not sharply defined, and a single integer score combined with a multiplicative risk priority number (RPN) cannot represent this uncertainty in sufficient detail. Conventional FMEA can also compress different S/O/D combinations into the same RPN and effectively treat the three factors as equally important [21,22,23,24]. By contrast, fuzzy-FMEA can express expert linguistic judgments as fuzzy numbers, thereby mitigating uncertainty and allowing the relative importance of S/O/D to be reflected when needed. Recent studies have shown that fuzzy-FMEA can improve risk-priority interpretation in process systems where chemical, thermal, electrical, and operational hazards overlap, including rotary kiln-based activated carbon manufacturing [25,26].

Therefore, in this study, a laboratory-scale solvent-based electrospinning process was decomposed into 14 steps, and one representative failure mode was defined for each step. Conventional FMEA and fuzzy weighted geometric mean (FWGM)-based fuzzy-FMEA were then applied in parallel. The identified high-risk group was further linked to safety barriers to examine changes in risk before and after improvement scenarios. The contribution of this study lies not in proposing a new fuzzy aggregation algorithm, but in presenting a barrier-oriented application framework that links the hazard structure specific to electrospinning with conventional FMEA, FWGM-based fuzzy-FMEA, and safety-barrier prioritization [21,26,27,28]. Ultimately, the proposed framework is intended to support the identification of high-risk nodes in solvent-based nanofiber manufacturing and to inform standard operating procedures (SOPs), equipment improvement, and the prioritization of safety barriers. Specifically, the novelty lies in integrating step-specific electrospinning failure modes, hazard-type-specific S/O/D weighting, and barrier-package-oriented post-control interpretation within a single workflow for solvent-based electrospinning.

## 2. Materials and Methods

### 2.1. Study System and Process Scope

**Figure 1:**
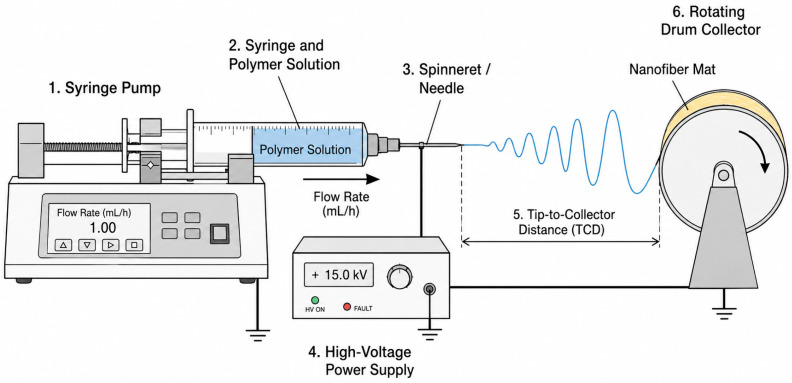
Schematic of the representative laboratory-scale solvent-based electrospinning system, including the syringe pump, polymer-solution syringe, metal spinneret/needle, high-voltage power supply, tip-to-collector region, rotating drum collector, and grounding points. The needle-to-collector region is treated as the main high-voltage and potential arcing/ignition zone, and the system is assumed to be operated inside a chemical fume hood or grounded closed enclosure with ventilation, access control/interlock protection, and discharge verification before troubleshooting or maintenance.

This study considered a laboratory-scale solvent-based electrospinning system as the target of analysis. The system was assumed to be a typical solution-electrospinning setup composed of a syringe pump, a metal needle, a high-voltage power supply, a metal collector, and either a chemical fume hood or a closed enclosure. A schematic of the overall configuration is shown in Figure 1. The analysis scope was divided into 14 steps following the actual experimental workflow, from material review/approval to maintenance.

Although multiple detailed failure modes can exist within each step, this study selected one representative failure mode for each step in order to clearly highlight the dominant risk and safety-barrier priority at the process-step level. Representative failure modes were selected by jointly considering the severity of potential consequences, the likelihood of direct operator intervention, the degree of direct linkage to electrical and chemical energy sources, and the vulnerability of existing safety barriers. Accordingly, the representative failure mode for each step should be interpreted not as a complete substitute for all detailed failure modes within that step, but as a reference scenario intended to reveal the dominant risk and priority target for improvement. The resulting process steps and representative failure modes are summarized in Table 1. Examples of additional within-step failure modes that could be incorporated in a more detailed decomposition are provided in Supplementary Table S1.

### 2.2. Expert Panel, Evaluation Procedure, and Scoring Principles

The S/O/D ratings and hazard-type-specific weights used in this study were determined by a five-member expert panel. The panel consisted of one chemical engineering researcher specializing in nanomaterials, one safety engineering researcher, one electrical engineering researcher, one laboratory safety specialist, and one health specialist. All participants held doctoral degrees and had more than 10 years of relevant research or professional experience. This small multidisciplinary expert-panel configuration is consistent with common practice in process safety and fuzzy-FMEA studies and enables the simultaneous consideration of process technology, electrical safety, exposure health, laboratory operation, and environment, health, and safety (EHS) perspectives [20,21,27,28].

The evaluation procedure comprised the following steps: (1) pre-alignment of process scope, terminology, and the S/O/D criteria; (2) review of the causes, consequences, and current safety barriers for each failure mode; (3) independent rating by each expert; (4) a consensus meeting for items showing divergence; and (5) final confirmation of the consensus scores and hazard-type weights. The same rating sheet and evidence criteria were applied to all failure modes, and the same final S/O/D set was used for both conventional FMEA and fuzzy-FMEA. Accordingly, differences between the two methods can be interpreted as differences in prioritization logic rather than differences in evaluator composition.

To clarify the consensus-building process, divergent items were defined as cases in which the range of expert scores was two points or greater in any S/O/D dimension or in which the hazard-type classification differed among experts. These items were re-discussed in a moderated consensus meeting, and outlier ratings were not automatically discarded; they were retained when supported by a credible accident-escalation pathway, high-voltage accessibility, solvent-exposure potential, or safety-barrier vulnerability. Otherwise, the final consensus score was determined based on the median tendency and the agreed evidence-based interpretation. The procedure was not designed as a formal multi-round Delphi survey; therefore, it is described as a structured consensus process rather than a formal Delphi method. Formal inter-rater agreement statistics were not calculated; instead, pre-consensus dispersion was used to identify items requiring reconciliation. A summary of the consensus procedure and final consensus scores is provided in Supplementary Table S2.

Scoring was based on a conservative interpretation, and adjustments were allowed only when an existing or proposed safety barrier could realistically reduce occurrence (O) or improve detection (D). Hazard-type-specific weights were established in a similar manner: each expert independently evaluated the relative importance of S/O/D using the linguistic scales very high (VH), high (H), and medium (M), after which the final values were adjusted through consensus. As a result, the rating logic adopted in this study was not a simple risk-impression exercise, but a procedure that reflected both the accident-escalation pathways expected in the absence of safety barriers and the interruption effects expected when barriers are present.

### 2.3. FMEA Scoring Criteria

In this study, severity (S), occurrence (O), and detection (D) were evaluated on a five-point scale according to the basic conventional FMEA framework, as summarized in Table 2 [20,21]. Severity reflected potential effects on human health, the possibility of localized fire, electric shock, or equipment damage, and the extent to which the hazard could propagate through the process. Occurrence reflected the likelihood of occurrence during routine laboratory activity, the frequency of troubleshooting, operational instability, and the repetitiveness of exposure opportunities. Detection was defined on the basis of the ability of safety barriers to identify a failure mode proactively before an accident occurred.

### 2.4. Conventional FMEA and Auxiliary Comparison Index

The risk priority number (RPN) for conventional FMEA was calculated using Equation (1). In addition, for visual comparison with the fuzzy risk priority number (FRPN), a normalized RPN (RPN_norm_) was calculated using the min–max transformation shown in Equation (2) to rescale RPN to a 0–100 range. This auxiliary index was used only as a simple comparison metric to show how failure modes within the same RPN group were re-ordered in fuzzy-FMEA rather than as an absolute method-to-method comparison [20,21].

(1) $$RPN_{i}=S_{i}\times O_{i}\times D_{i}$$

(2) $$RPN_{norm,i}=100\times \frac{RPN_{i}-RPN_{min}}{RPN_{max}-RPN_{min}}$$

### 2.5. FWGM-Based Fuzzy-FMEA

FWGM-based fuzzy-FMEA was adopted in this study because it enables the derivation of a continuous risk indicator that remains directly comparable with the conventional 1–5 FMEA framework while reflecting the hazard-type specificity of electrospinning processes. In other words, rather than increasing the complexity of fuzzy logic itself, this study focused on explicitly weighting S/O/D according to process-specific hazard characteristics [21,25,26].

First, the integer S/O/D scores were converted into triangular fuzzy numbers (TFNs), and the 1–5 conversion scheme used for this purpose is summarized in Table 3. Next, each failure mode was classified as chemical-dominant, electrical-dominant, fire/static-dominant, or combined-dominant, and different relative weights were assigned to S/O/D for each hazard type [21,25,26].

The hazard-type-specific linguistic weights and normalized S/O/D weights are summarized in Table 4. Here, very high (VH), high (H), and medium (M) denote the relative importance of each dimension. These linguistic weights were first mapped to predefined triangular fuzzy numbers, after which representative values were calculated and normalized by the sum of S/O/D to derive the numerical weights. The normalized weights are reported to three decimal places for readability, whereas the FRPN calculations used the internal unrounded values. FRPN was then calculated using Equations (3)–(6). Equation (3) represents the fuzzy weighted geometric mean, Equation (4) the weighted triangular fuzzy result, Equation (5) the defuzzification step, and Equation (6) the conversion to a 100-point scale. Here, $w_{S}$, $w_{O}$, and $w_{D}$ denote the normalized weights for severity, occurrence, and detection, respectively, as listed in Table 4. In Equation (4), $O_{il}$, $O_{im}$, $O_{iu}$, $S_{il}$, $S_{im}$, $S_{iu}$, $D_{il}$, $D_{im}$, and $D_{iu}$ denote the lower, modal, and upper values of the TFNs corresponding to the S/O/D scores of the i-th failure mode. The values il, im, and iu obtained from Equation (4) represent the three vertices of the aggregated FRPN TFN for the i-th failure mode; $FRPN_{i}^{⋆}$ in Equation (5) is the defuzzified representative value, and $FRPN_{i,100}$ in Equation (6) is the final FRPN converted to a 100-point scale. Here, i denotes the index of the representative failure mode corresponding to each process step in Table 1, and i = 1, …, 14 in this study [21,25,26,27].

The linguistic-weight TFNs used for normalization were M = (0.3, 0.5, 0.7), H = (0.5, 0.7, 0.9), and VH = (0.7, 0.9, 1.0), with centroid representative values of 0.500, 0.700, and 0.867, respectively. For example, the H/H/M chemical-dominant structure yields 0.7/(0.7 + 0.7 + 0.5) = 0.368, 0.7/(0.7 + 0.7 + 0.5) = 0.368, and 0.5/(0.7 + 0.7 + 0.5) = 0.263. Further definitions, full derivations, and the rationale for the hazard-type-specific S/O/D structures are provided in Supplementary Tables S3 and S4, and a worked FRPN calculation is provided in Supplementary Table S5.

The within-tie implementation priority criteria were predefined during the pre-alignment stage and were used only when exact FRPN ties occurred. When identical FRPN values were obtained, no additional numerical model was introduced; instead, within-tie implementation priority criteria were used to distinguish the order of action among items in the same rank group. Within-tie implementation priority was additionally interpreted based on (i) the possibility of direct intervention under energized or residual-energy conditions, (ii) the absence of independent engineering barriers, and (iii) the potential for immediate escalation to electric shock, arcing, or ignition.

(3) $${\tilde{FRPN}}_{i}={\tilde{S}}_{i}^{w_{S}}⊗{\tilde{O}}_{i}^{w_{O}}⊗{\tilde{D}}_{i}^{w_{D}}$$

(4) $${\tilde{FRPN}}_{i}=\left(l_{i},m_{i},u_{i}\right)\;l_{i}=S_{il}^{w_{S}}O_{il}^{w_{O}}D_{il}^{w_{D}}\;m_{i}=S_{im}^{w_{S}}O_{im}^{w_{O}}D_{im}^{w_{D}}\;u_{i}=S_{iu}^{w_{S}}O_{iu}^{w_{O}}D_{iu}^{w_{D}}$$

(5) $$FRPN_{i}^{⋆}=\frac{l_{i}+m_{i}+u_{i}}{3}$$

(6) $$FRPN_{i,100}=20\times FRPN_{i}^{⋆}$$

**Figure 2:**
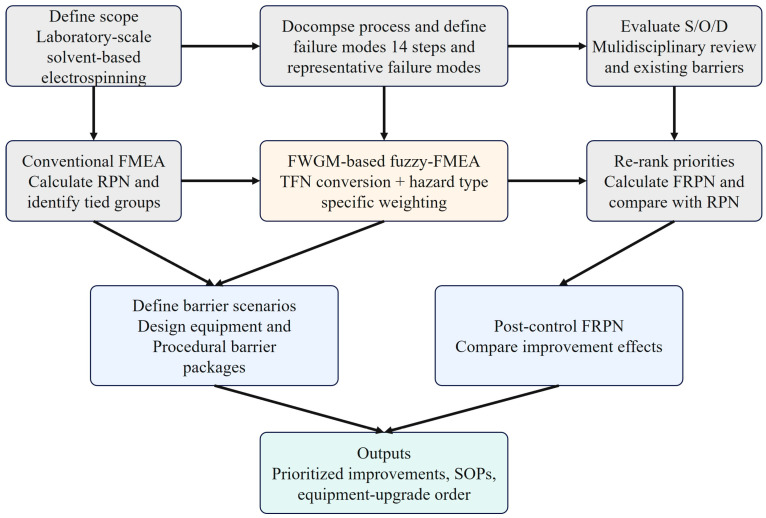
Analytical workflow of the barrier-oriented FWGM-based fuzzy-FMEA for electrospinning process risk assessment.

Subsequent safety-barrier scenario analysis was performed for the highest-ranked FRPN failure modes and representative failure modes with high barrier sensitivity. For each failure mode, an engineering and managerial safety-barrier package was first defined, and only the S/O/D dimensions directly affected by the barrier were re-scored. In general, severity (S) was retained unless the accident consequence itself would change; occurrence (O) was adjusted only when actual occurrence likelihood could be reduced through lower exposure opportunity, prevention of operator error, or elimination of ignition sources; and detection (D) was adjusted only when new barriers such as interlocks, checklists, indicators, or verification procedures increased the likelihood of identifying the failure mode before an accident. The revised S/O/D values were then re-entered using the same hazard-type weights and Equations (3)–(6) to calculate post-control FRPN, which was compared with the pre-control value [21,27]. Finally, the overall analysis procedure was summarized in the workflow shown in Figure 2.

## 3. Results and Discussion

### 3.1. Stepwise Risk Structure and Conventional FMEA Results

Conventional FMEA indicated that the highest-risk nodes were concentrated in preparation and intervention steps rather than only during voltage application. The RPN 60 group comprised material review/approval, equipment setup, pre-start inspection, and response to abnormalities (Table 5). This pattern shows that upstream information control, high-voltage accessibility, pre-start confirmation, and abnormal-response intervention activities are major contributors to electrospinning risk.

Rather than restating each score, the discussion focuses on mechanism-level interpretation. Material review/approval functions as an upstream latent hazard because missing SDS or additive information can propagate to PPE, ventilation, and waste-handling decisions. Equipment setup primarily reflects exposure to accessible high-voltage components due to inadequate grounding, shielding, or interlock protection, whereas response to abnormalities involves direct operator intervention during troubleshooting under energized or residual-energy conditions. Pre-start inspection and voltage application/start-up represent combined chemical–electrical nodes where ventilation, spacing, door status, and initial flow conditions determine whether flammable vapor and ignition sources become simultaneously active. These differences explain why conventional FMEA provides a useful screening overview but is limited in distinguishing electrical, chemical, and ignition mechanisms within tied RPN groups.

### 3.2. Risk-Priority Re-Ranking Using FWGM-Based Fuzzy-FMEA

FWGM-based fuzzy-FMEA decomposed several conventional RPN ties by incorporating fuzzy score ranges and hazard-type-specific S/O/D weights (Table 6). The RPN 60 group was redistributed into a narrower FRPN range, while equipment setup and response to abnormalities remained tied because they shared the same S/O/D vector and electrical-dominant weight structure. This result indicates that FWGM-based fuzzy-FMEA reduces, but does not eliminate, ranking redundancy when identical inputs are assigned [21,26,27].

Across the lower RPN groups, FRPN reordering reflected differences in dominant hazard mechanisms rather than merely numerical score products. For example, solution preparation and voltage application/start-up were separated despite belonging to the same conventional RPN group because the former primarily represents a chemically driven exposure hazard, whereas the latter represents a combined chemical–electrical ignition scenario involving dripping, leakage, arcing, and localized ignition risk. Similarly, electrical-intervention modes such as maintenance, shutdown/discharge, and normal operation were prioritized differently from fire/static and waste-handling modes, even when conventional RPN values were identical. These results suggest that fuzzy-FMEA can reveal differences in underlying hazard mechanisms that remain obscured when risks are represented solely by multiplicative RPN values.

**Figure 3:**
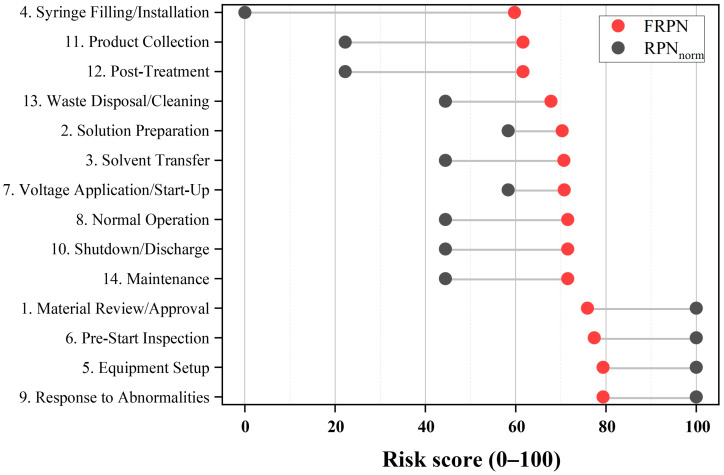
Re-ranking of risk priorities by comparing normalized RPN and FRPN.

Figure 3 provides a visual summary of the re-ranking behavior by comparing normalized RPN and FRPN results, illustrating how failure modes compressed into the same conventional RPN groups become redistributed according to hazard-specific weighting and fuzzy aggregation. Accordingly, Table 6 presents the detailed FRPN values together with the corresponding within-tie implementation order.

Within-tie implementation priority was interpreted using predefined practical criteria established during the pre-alignment stage: direct intervention under energized or residual-energy conditions, absence of independent engineering barriers, and potential for immediate escalation to electric shock, arcing, or ignition. These criteria do not replace the numerical FRPN itself; rather, they clarify the implementation order of safety barriers within the same FRPN group. For example, within the highest-FRPN tie group, response to abnormalities was assigned a higher implementation priority than equipment setup because troubleshooting activities may require direct intervention under energized or residual-energy conditions. Therefore, the within-tie criteria should be interpreted as a supplementary implementation guide for barrier prioritization rather than an alternative ranking method.

### 3.3. Engineering Interpretation of High-Risk Failure Modes and Safety-Barrier Prioritization

When the highest-ranking FRPN nodes are interpreted from an engineering safety perspective, the dominant risk structure of the electrospinning process can be summarized along four axes, as presented in Table 7. The first is an upstream information-based hazard, in which failure in material review/approval fixes inappropriate safety conditions for the entire process. The second is a direct high-voltage access hazard, in which direct access during equipment setup or normal operation creates electric shock and arcing risks. The third is a pre-start combined hazard, in which electrical and chemical risks are coupled the moment voltage is applied without confirming ventilation, door status, and spacing. The fourth is an abnormal-response intervention hazard, in which operator intervention during response to abnormalities and maintenance readily encounters stored energy.

This interpretation has implications beyond simple ranking. For example, the key improvement measure for equipment setup is not stronger PPE, but engineering barriers such as a grounded enclosure, interlocks, and grounding continuity verification. By contrast, material review/approval requires information-based barriers such as an approval gate, review of composition changes, and SDS review. Pre-start inspection and voltage application/start-up primarily depend on procedural barriers such as operating limits, door-closed status, ventilation confirmation, and checklists, while response to abnormalities should prioritize de-energize-discharge-verify procedures and troubleshooting SOPs. Thus, even among high-risk nodes, the required safety-barrier types differ. Revealing such differences is the practical value of barrier-oriented fuzzy-FMEA. In particular, the use of enclosures and interlocks during equipment setup and the management of operating windows during pre-start inspection and voltage application/start-up may help improve the stable operation of nanofiber manufacturing by prioritizing worker safety while also supporting jet stability and solvent-vapor control.

### 3.4. Risk Reduction Effect of Safety-Barrier Application Scenarios

The post-control scenario analysis was reorganized to emphasize barrier logic rather than repeat the numerical values listed in Table 8. The detailed post-control recalculation sheet is provided in Supplementary Table S6. Barrier packages were defined according to dominant hazard mechanisms: grounded enclosures, interlocks, grounding-continuity checks, de-energize-discharge-verify procedures, and troubleshooting SOPs for electrical-intervention hazards; and bonding/grounding, pre-start checklists, sealed transfer and waste-handling controls, and ventilation-related controls for ignition and chemical hazards.

The largest reductions were obtained for failure modes in which the barrier package simultaneously reduced occurrence and improved detection, particularly response to abnormalities and equipment setup. The largest reductions were observed for these failure modes because the corresponding barrier packages reduced the likelihood of operator intervention under energized conditions while improving early detection through interlocks, inspections, and verification procedures. Smaller but still meaningful reductions were observed for material review/approval, pre-start inspection, and solvent transfer, reflecting the fact that information-based and managerial barriers generally influence exposure probability and detectability rather than intrinsic consequence severity.

**Figure 4:**
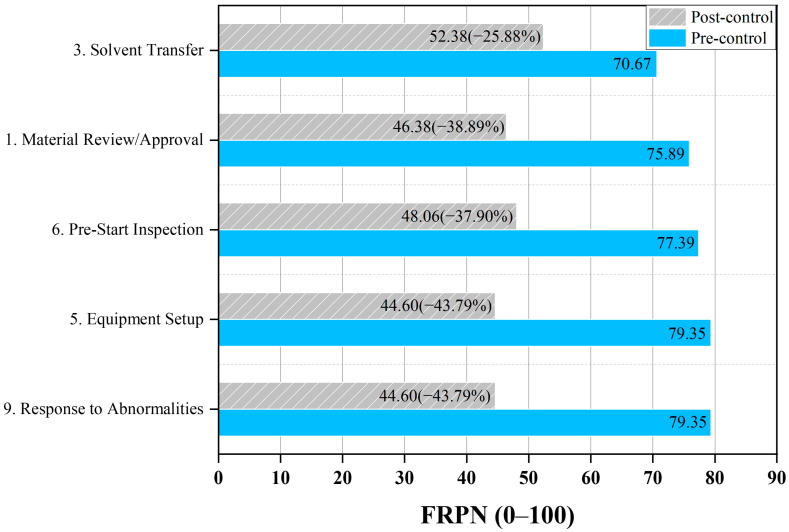
FRPN before and after application of safety barriers for prioritized failure modes.

Accordingly, Figure 4 and Table 8 report the quantitative pre-/post-control FRPN values, whereas the text emphasizes why different accident mechanisms respond differently to safety barriers. Maintenance-related risk reduction was interpreted together with the electrical-intervention barrier package because de-energization and isolation-verification measures substantially overlap with those applied to response-to-abnormality and shutdown/discharge scenarios. This interpretation supports a package-based improvement strategy rather than repeated application of a single control across all process steps.

The post-control FRPN values should therefore be interpreted as scenario-based estimates rather than measurement-based validation. Nevertheless, the proposed barrier packages are consistent with common laboratory EHS principles: fume hoods are recommended for work involving flammable or toxic chemicals; bonding and grounding are used to reduce static charge accumulation during flammable-liquid transfer; and high-voltage electrospinning should incorporate grounded enclosures, interlocks, access control, and discharge-verification steps before troubleshooting or maintenance [29,30,31,32].

### 3.5. Study Limitations and Future Research Directions

This study focused on presenting FWGM-based fuzzy-FMEA results for the reference scenario and the improvement effect of representative safety-barrier packages; therefore, an exhaustive robustness analysis across TFN widths, alternative defuzzification schemes, and simultaneous weight variations was outside the intended scope. A limited one-at-a-time sensitivity check for the high-priority RPN 60 group was included in Supplementary Table S7 to examine local stability under ±10% weight perturbation. In addition, the safety-barrier effects presented here are based on scenario-based post-control recalculation and do not include measurement-based validation such as actual exposure concentration, leakage current, arc frequency, or reductions in near-miss events. Therefore, the FRPN rankings reported in this study should be interpreted as priorities derived from the reference scenario and consensus expert ratings, and the sensitivity to fuzzy parameter selection and the actual performance of the proposed barrier packages remain subjects for future validation.

Several structural limitations should also be noted. First, because the analysis target is a typical laboratory-scale solvent-based electrospinning system, it does not fully encompass the equipment complexity of multi-nozzle, pilot-scale, or continuous-production systems. Second, because a single representative failure mode was used for each process step, within-step variation was compressed. Third, because the hazard-type weights and post-control score adjustments were based on the independent judgments and consensus of a five-member expert panel, the model cannot be generalized as a universally validated framework without larger panels or inter-institutional validation. Fourth, when identical input vectors and identical weights produced tied FRPN values, this study interpreted them using within-tie implementation priority criteria; however, these criteria are intended only to interpret implementation order within tied FRPN groups and do not constitute a new numerical model.

The present Supplementary Materials provide examples of additional within-step failure modes, the expert consensus summary, linguistic-weight definitions and derivations, a worked FRPN calculation, post-control recalculation tables, and a limited sensitivity check. Future studies should extend these materials by incorporating larger anonymized expert-rating datasets and by conducting robustness analyses on TFN width, defuzzification methods, and weight systems [27,28]. It would also be worthwhile to examine recent extensions such as interval type-2 fuzzy-FMEA, D-number-based and fuzzy-integral FMEA approaches, integrated MCDM-FMEA frameworks, and other advanced uncertainty-based risk-assessment approaches to enhance uncertainty representation, robustness, and external verifiability [33,34,35,36,37,38,39].

## 4. Conclusions

This study applied conventional FMEA and FWGM-based fuzzy-FMEA in parallel to a laboratory-scale solvent-based electrospinning process in order to interpret process risk step by step and connect the results to safety-barrier prioritization.

First, conventional FMEA showed that the major risks in electrospinning are concentrated not only at the moment of voltage application but also in preparation and intervention steps such as material review/approval, equipment setup, pre-start inspection, and response to abnormalities.

Second, fuzzy-FMEA subdivided tied RPN groups and re-ranked failure modes with high-voltage accessibility and potential intervention under stored-energy conditions into the upper risk group. By combining this with within-tie implementation priority criteria, the study proposed the practical action order of response to abnormalities (9), equipment setup (5), pre-start inspection (6), and material review/approval (1).

Third, barrier-oriented interpretation structured four dominant mechanisms—upstream information-based hazards, direct high-voltage access hazards, pre-start combined hazards, and intervention during abnormal situations—and proposed corresponding engineering and procedural safety-barrier packages.

Fourth, the scenario-based post-control analysis showed that safety barriers such as grounded enclosures, interlocks, de-energize-discharge-verify procedures, pre-start checklists, and bonding/grounding can substantially lower FRPN for high-risk failure modes, with reductions of approximately 43.79% for both equipment setup and response to abnormalities.

Overall, the main contribution of this study is not the proposal of a new mathematical fuzzy aggregation model, but the presentation of a barrier-oriented application framework that interprets electrospinning as both a nanofiber manufacturing process and a chemical–electrical composite hazard system and connects fuzzy-FMEA results with practical safety-barrier packages. This framework can support SOP development, equipment improvement, training prioritization, and the preparation of institutional risk-assessment documents. With future incorporation of electrospinning-specific hazard factors, it may be extended into a more sophisticated process-safety evaluation model that addresses both stable operation and safety in materials-manufacturing processes.

## Figures and Tables

**Table 1:** Process steps and representative failure modes in electrospinning.

No.	Process Step	Representative Failure Mode
1	Material Review/Approval	Failure to review the safety data sheet (SDS) or omission of additives, resulting in underestimated hazard
2	Solution Preparation	Preparation outside the hood or prolonged open preparation
3	Solvent Transfer	Transfer of flammable solvent without bonding/grounding
4	Syringe Filling/Installation	Leakage or splashing during syringe/needle connection
5	Equipment Setup	Poor collector grounding, exposed high-voltage parts, and absence of interlocks
6	Pre-Start Inspection	Start-up without confirming exhaust, door status, or distance
7	Voltage Application/Start-Up	Dripping/arc generation due to excessive voltage, excessive flow rate, or improper distance
8	Normal Operation	Direct adjustment of the needle/collector during energization
9	Response to Abnormalities	Clog removal without shutdown and discharge confirmation
10	Shutdown/Discharge	Contact under residual-charge condition after power-off
11	Product Collection	Collection of a wet fiber mat on an open bench
12	Post-Treatment	Immediate heat treatment of samples with residual solvent
13	Waste Disposal/Cleaning	Open waste containers and unattended contaminated materials
14	Maintenance	Opening the power supply or using damaged cables while power remains connected

**Table 2:** FMEA scoring criteria.

Score	Severity (S)	Occurrence (O)	Detection (D)
1	Mild housekeeping-level problem; no health impact	Rare under normal operating conditions	Almost certainly detectable in advance by a checklist, interlock, indicator light, etc.
2	Minor exposure or localized contamination; first-aid level	May occur intermittently under abnormal or maintenance conditions	Detectable with high probability through an inspection procedure or operator observation
3	Reversible health effect, small spill, temporary process interruption	May occasionally occur during routine laboratory activity	Detectable depending on operator skill, but omissions are possible
4	Serious exposure, localized fire, electrical burn, or equipment damage	May occur frequently under unstable operating conditions	Likely to be detected only after the abnormality has progressed
5	Major accident, severe electric shock, fire/explosion, or fatal toxic exposure	Repetitive or nearly inevitable in the absence of controls	Very difficult to detect before an imminent accident

**Table 3:** Conversion of integer scores to triangular fuzzy numbers.

Integer Score	Triangular Fuzzy Number (TFN)
1	(1, 1, 2)
2	(1, 2, 3)
3	(2, 3, 4)
4	(3, 4, 5)
5	(4, 5, 5)

**Table 4:** Hazard-type-specific linguistic weights and normalized S/O/D weights.

Hazard Type	Linguistic Weights (S, O, D)	Normalized Weights ($w_{S}$, $w_{O}$, $w_{D}$)	Applied Steps
Chemical-dominant	H, H, M	(0.368, 0.368, 0.263)	Material Review/Approval (1), Solution Preparation (2), Syringe Filling/Installation (4), Product Collection (11), Post-Treatment (12)
Electrical-dominant	VH, M, H	(0.419, 0.242, 0.339)	Equipment Setup (5), Normal Operation (8), Response to Abnormalities (9), Shutdown/Discharge (10), Maintenance (14)
Fire/static-dominant	VH, M, M	(0.464, 0.268, 0.268)	Solvent Transfer (3)
Combined-dominant	VH, H, H	(0.383, 0.309, 0.309)	Pre-Start Inspection (6), Voltage Application/Start-Up (7), Waste Disposal/Cleaning (13)

**Table 5:** Conventional FMEA results for the electrospinning process.

No.	Process Step	Failure Mode	Main Cause	Potential Effect	Hazard Type	S/O/D	RPN
1	Material Review/Approval	Failure to review the safety data sheet (SDS) or omission of additives	Use of customary recipes, supplier change, omission of additive/nanofiller review	Inappropriate PPE and ventilation, underestimated toxicity/flammability, process-wide risk propagation	Chemical-dominant	5/3/4	60
2	Solution Preparation	Preparation outside the hood or prolonged open preparation	Open-bench work, prolonged container opening, prioritization of convenience	Solvent-vapor exposure, local vapor accumulation, increased flammability risk	Chemical-dominant	5/3/3	45
3	Solvent Transfer	Transfer of flammable solvent without bonding/grounding	Lack of bonding/grounding procedure, inadequate electrostatic control	Electrostatic spark ignition, fire, secondary worker exposure	Fire/static-dominant	5/2/4	40
4	Syringe Filling/Installation	Leakage or splashing during syringe/needle connection	Overfilling, incomplete Luer connection, forced injection of high-viscosity solution	Skin/eye exposure, contamination, localized vapor release	Chemical-dominant	4/3/2	24
5	Equipment Setup	Poor collector grounding, exposed high-voltage parts, and absence of interlocks	Loose grounding wire, homemade apparatus, insulation deterioration, inadequate shielding	Electric shock, arcing, fire, equipment damage	Electrical-dominant	5/3/4	60
6	Pre-Start Inspection	Start-up without confirming exhaust, door status, or distance	Absence of a pre-start checklist, rushed operation, and door status not checked	Ignition risk, electric shock, defective fiber formation	Combined-dominant	5/3/4	60
7	Voltage Application/Start-Up	Dripping/arc generation due to excessive voltage, excessive flow rate, or improper distance	Unverified start-up conditions, recipe variation, and error in initial settings	Leakage, fiber defects, arcing, localized fire	Combined-dominant	5/3/3	45
8	Normal Operation	Direct adjustment of the needle/collector during energization	Ad hoc intervention for process stabilization, insufficient access restriction	Direct electric shock, secondary injury	Electrical-dominant	5/2/4	40
9	Response to Abnormalities	Clog removal without shutdown and discharge confirmation	Insufficient understanding of residual charge, absence of a troubleshooting SOP	Electric shock, arc, solution splashing, facial/hand exposure	Electrical-dominant	5/3/4	60
10	Shutdown/Discharge	Contact under residual-charge condition after power-off	Mistaken belief that power-off means safe, absence of a discharge verification procedure	Electric shock, arc, secondary accident during shutdown	Electrical-dominant	5/2/4	40
11	Product Collection	Collection of a wet fiber mat on an open bench	Insufficient confirmation of drying, handling outside the hood	Residual-solvent exposure, contamination spread	Chemical-dominant	4/2/4	32
12	Post-Treatment	Immediate heat treatment of samples with residual solvent	Insufficient drying time, absence of criteria, failure to review equipment suitability	Vapor release, possible fire in the oven, increased exposure	Chemical-dominant	4/2/4	32
13	Waste Disposal/Cleaning	Open waste containers and unattended contaminated materials	Stored near equipment for convenience, lid not fully closed	Vapor accumulation, secondary ignition, persistent exposure	Combined-dominant	5/2/4	40
14	Maintenance	Opening the power supply or using damaged cables while power remains connected	Unauthorized repair, absence of regular inspection, and damaged cable left unaddressed	Severe electric shock, arc, fire, reduced equipment reliability	Electrical-dominant	5/2/4	40

**Table 6:** Fuzzy-FMEA results and within-tie implementation priority for the electrospinning process.

Rank Group (Implementation Priority)	No.	Process Step	Hazard Type	S/O/D	Conventional RPN	FRPN (TFN)	FRPN (0–100)
1T(1)	9	Response to Abnormalities	Electrical-dominant	5/3/4	60	(3.068, 4.097, 4.737)	79.35
1T(2)	5	Equipment Setup	Electrical-dominant	5/3/4	60	(3.068, 4.097, 4.737)	79.35
3(3)	6	Pre-Start Inspection	Combined-dominant	5/3/4	60	(2.955, 3.986, 4.667)	77.39
4(4)	1	Material Review/Approval	Chemical-dominant	5/3/4	60	(2.873, 3.906, 4.605)	75.89
5T(5)	14	Maintenance	Electrical-dominant	5/2/4	40	(2.595, 3.714, 4.419)	71.52
5T(6)	10	Shutdown/Discharge	Electrical-dominant	5/2/4	40	(2.595, 3.714, 4.419)	71.52
5T(7)	8	Normal Operation	Electrical-dominant	5/2/4	40	(2.595, 3.714, 4.419)	71.52
8(8)	7	Voltage Application/Start-Up	Combined-dominant	5/3/3	45	(2.608, 3.648, 4.357)	70.76
9(9)	3	Solvent Transfer	Fire/static-dominant	5/2/4	40	(2.554, 3.685, 4.361)	70.67
10(10)	2	Solution Preparation	Chemical-dominant	5/3/3	45	(2.582, 3.621, 4.343)	70.30
11(11)	13	Waste Disposal/Cleaning	Combined-dominant	5/2/4	40	(2.385, 3.517, 4.270)	67.81
12T(12)	12	Post-Treatment	Chemical-dominant	4/2/4	32	(2.001, 3.099, 4.142)	61.62
12T(13)	11	Product Collection	Chemical-dominant	4/2/4	32	(2.001, 3.099, 4.142)	61.62
14(14)	4	Syringe Filling/Installation	Chemical-dominant	4/3/2	24	(1.935, 2.998, 4.026)	59.73

**Table 7:** Engineering characteristics of major high-risk failure modes and prioritized safety barriers.

Process Step	Safety Engineering Characteristic	Engineering Root Cause	Prioritized Safety-Barrier Package
Material Review/Approval (1)	Upstream information-based hazard	No SDS review, omitted composition, absence of an approval system	Mandatory SDS review, re-approval of composition changes, pre-review system
Equipment Setup (5)	Direct high-voltage access hazard	Poor grounding, exposed high-voltage parts, absence of an enclosure/interlock	Grounded enclosure, interlock, grounding continuity check
Pre-Start Inspection (6)	Pre-start combined hazard	Ventilation/door/distance not checked, absence of a pre-start procedure	Pre-start checklist, ventilation confirmation, door-closed confirmation
Voltage Application/Start-Up (7)	Coupled operational instability and ignition hazard	Excessive voltage, excessive flow rate, improper distance, erroneous initial conditions	Validated operating window, focused initial observation, drip tray
Response to Abnormalities (9)	Abnormal-response intervention hazard	Access before discharge, absence of a troubleshooting SOP	De-energize-discharge-verify procedure, access control
Solvent Transfer (3)	Electrostatic ignition hazard	Bonding/grounding not implemented	Bonding/grounding, pre-transfer check
Shutdown/Discharge (10)	Residual-charge contact hazard	Misconception that power-off means safe, absence of discharge confirmation	Residual-charge confirmation, shutdown checklist
Maintenance (14)	Maintenance without de-energization confirmation hazard	Failure to comply with power isolation, damaged cable left unaddressed	De-energized maintenance principle, periodic inspection, and replacement of damaged cables

**Table 8:** Post-control FRPN results for prioritized failure modes.

Process Step	Key Safety Barrier	Pre-Control FRPN	Post-Control FRPN	Reduction Rate (%)	Main Dimension of Change
Response to Abnormalities (9)	De-energize-discharge-verify, troubleshooting SOP, access control	79.35	44.60	43.79	Mostly improved O and D
Equipment Setup (5)	Grounded enclosure, door interlock, grounding continuity check	79.35	44.60	43.79	Mostly improved O and D
Pre-Start Inspection (6)	Pre-start checklist, exhaust/door confirmation, validated operating window	77.39	48.06	37.90	Mostly improved O and D
Material Review/Approval (1)	SDS review gate, composition re-approval, change review	75.89	46.38	38.89	Mostly improved O and D
Solvent Transfer (3)	Bonding/grounding, sealed transfer, transfer checklist	70.67	52.38	25.88	Mostly improved O and D

Note: Maintenance (14) was not separately tabulated because its post-control logic substantially overlapped with the electrical-intervention barrier scenarios represented by response to abnormalities and shutdown/discharge.

## Data Availability

The original contributions presented in this study are included in the article/Supplementary Materials. Further inquiries can be directed to the corresponding author.

## Supplementary Materials

The following supporting information can be downloaded at: https://www.mdpi.com/article/10.3390/ma19122673/s1, Table S1: Examples of additional failure modes that could be considered in a more detailed decomposition; Table S2: Expert consensus procedure and anonymized scoring summary; Table S3: Triangular fuzzy number definitions for linguistic weights; Table S4: Derivation of hazard-type-specific normalized S/O/D weights and rationale; Table S5: Worked FRPN calculation example for Response to Abnormalities (Step 9); Table S5 continued: Baseline FRPN calculation sheet for all representative failure modes; Table S6: Post-control recalculation sheet for prioritized failure modes; Table S7: Summary of one-at-a-time sensitivity analysis for the high-priority RPN 60 group; Table S7 continued: Detailed one-at-a-time perturbation results.
