# Supplementary material for: Barrier-Oriented FWGM-Based Fuzzy-FMEA for Risk Assessment and Safety-Barrier Prioritization in Solvent-Based Electrospinning Processes
Source: Materials (Basel). 2026 Jun 22;19(12):2673. doi: 10.3390/ma19122673 (PMC13303637; doi:10.3390/ma19122673)
Supplement: Supplementary file 1 [file materials-19-02673-s001.zip › materials-4334809-supplementary.pdf]

Supplementary Materials

Barrier-Oriented FWGM-Based Fuzzy-FMEA for Risk Assessment and Safety-Barrier Prioritization in Solvent-Based Electrospinning Processes

Jong Gu Kim[1], Byong Chol Bai[2,*]

[1]Department of Fire and Disaster Prevention and Safety, Sun Moon University, Asan 31460, Republic of Ko-rea; jonggukim@sunmoon.ac.kr

[2]Department of Chemical Engineering, Daejin University, Pocheon 11159, Republic of Korea; baibc0820@daejin.ac.kr

*Correspondence: baibc0820@daejin.ac.kr

This supplementary material provides additional methodological details and calculation sheets corresponding to the revised manuscript. The table numbering follows the order of first citation in the main text: Table S1 through Table S7.

S1. Examples of additional within-step failure modes

Table S1. Examples of additional failure modes that could be considered in a more detailed decomposition.

No.	Process step	Example additional failure modes	Dominant hazards	How the framework would treat them
1	Material Review/Approval	Supplier SDS revision not checked; nanofiller additive toxicity not reviewed; incompatible solvent substitute approved by habit	Chemical exposure; flammability; upstream information error	Each case can be entered as a separate FMEA row with its own S/O/D and hazard type; priority could shift if a specific additive or solvent introduces higher toxicity or ignition risk.
2	Solution Preparation	Polymer weighing and mixing outside local exhaust; prolonged open vessel; hood sash not positioned correctly	Solvent vapor exposure; flammable vapor accumulation	Separate rows would distinguish chronic exposure from acute flammable-vapor accumulation.
3	Solvent Transfer	Ungrounded receiving container; splash during transfer; waste solvent decanting near energized equipment	Static ignition; dermal/eye exposure; secondary fire	A higher occurrence or poorer detection score could elevate the transfer step above some electrical maintenance items.
4	Syringe Filling/Installation	Luer-lock not secured; needle blocked during filling; overfilled syringe; high-viscosity solution forced through needle	Splashing; leakage; localized vapor release	Each mode can be weighted as chemical-dominant unless coupled with energized equipment.
5	Equipment Setup	Damaged high-voltage cable; collector ground loose; exposed connector; high-voltage lead positioned near solvent container	Electric shock; arcing; ignition source	Engineering-barrier sensitivity can be assessed separately for grounding, shielding, and interlock failures.
6	Pre-Start Inspection	Ventilation not confirmed; enclosure door open; tip-to-collector distance incorrect; collector ground not verified	Combined chemical/electrical hazard	Multiple pre-start omissions may yield different occurrence and detection values depending on checklist design.
7	Voltage Application/Start-Up	Initial voltage ramp too fast; excessive flow rate; wet droplet reaches collector; sudden arc during start-up	Arc; local fire; exposure to wet solution	Detailed decomposition would separate process-instability risk from ignition risk.
8	Normal Operation	Needle alignment adjusted by hand; fiber accumulation removed during energization; collector repositioned while energized	Direct electric shock; secondary injury	Direct-access modes would remain electrical-dominant and may rank high if intervention is frequent.
9	Response to Abnormalities	Needle clog removed before discharge; solution spill addressed near energized parts; abnormal noise/arcing investigated without shutdown	Stored-energy contact; splash; arc	These modes may remain top priority because abnormal-response tasks combine urgency with poor detection of residual energy.
10	Shutdown/Discharge	Assumption that power-off is safe; collector touched before discharge verification; discharge tool not used	Residual-charge electric shock	Detection score would strongly depend on whether discharge verification is mandatory and documented.
11	Product Collection	Wet mat removed outside hood; mat stored in open container; collector scraped without verifying solvent evaporation	Residual solvent inhalation; dermal exposure	Detailed modes would separate exposure during removal from exposure during storage.
12	Post-Treatment	Residual solvent sample placed in oven; incompatible heating equipment used; drying criterion not defined	Vapor release; oven fire; exposure	Severity may increase if heat treatment involves poorly ventilated equipment or ignition sources.
13	Waste Disposal/Cleaning	Open solvent waste bottle; contaminated wipes left near equipment; cleaning performed without ventilation	Vapor accumulation; secondary ignition; persistent exposure	Rows can be classified as combined-dominant when flammable vapor and nearby ignition sources coexist.
14	Maintenance	Unauthorized power-supply opening; damaged cable reused; maintenance performed without lockout/isolation verification	Severe electric shock; arc; fire	Detailed maintenance modes could rank high when damaged components are accessible or inspection intervals are long.

Note: These examples are illustrative. A more detailed FMEA can treat each additional failure mode as an independent row and apply the same conventional RPN and FWGM-based FRPN procedure.

S2. Expert consensus procedure and anonymized scoring summary

Table S2. Expert consensus procedure and anonymized scoring summary.

Item	Description
Panel composition	Five-member multidisciplinary panel: chemical engineering/nanomaterials, safety engineering, electrical engineering, laboratory safety, and health.
Independent pre-scoring	Each expert independently evaluated S/O/D using the same rating sheet and the same failure-mode definitions before consensus discussion.
Divergence screening	An item was treated as divergent if the range of expert scores was two points or greater in any S/O/D dimension, or if the hazard-type classification differed among experts.
Moderated reconciliation	Divergent items were re-discussed using accident pathway, high-voltage accessibility, solvent-exposure potential, existing safety barriers, SDS/EHS information, and process conditions.
Outlier handling	Outlier ratings were not automatically discarded. They were retained when supported by a credible escalation scenario; otherwise, the final score followed the median tendency and evidence-based consensus.
Use of final ratings	The same final consensus S/O/D set was used for conventional FMEA and FWGM-based fuzzy-FMEA, so differences between RPN and FRPN reflect prioritization logic rather than evaluator composition.
Confidentiality	The table below provides the anonymized final consensus scoring summary used for the calculations. Individual expert identities are not disclosed.

Note: The procedure was a structured consensus process with independent pre-scoring and moderated reconciliation. It was not described as a full multi-round Delphi survey.

No.	Process step	Hazard type	Final consensus S/O/D	Conventional RPN
1	Material Review/Approval	Chemical-dominant	5/3/4	60
2	Solution Preparation	Chemical-dominant	5/3/3	45
3	Solvent Transfer	Fire/static-dominant	5/2/4	40
4	Syringe Filling/Installation	Chemical-dominant	4/3/2	24
5	Equipment Setup	Electrical-dominant	5/3/4	60
6	Pre-Start Inspection	Combined-dominant	5/3/4	60
7	Voltage Application/Start-Up	Combined-dominant	5/3/3	45
8	Normal Operation	Electrical-dominant	5/2/4	40
9	Response to Abnormalities	Electrical-dominant	5/3/4	60
10	Shutdown/Discharge	Electrical-dominant	5/2/4	40
11	Product Collection	Chemical-dominant	4/2/4	32
12	Post-Treatment	Chemical-dominant	4/2/4	32
13	Waste Disposal/Cleaning	Combined-dominant	5/2/4	40
14	Maintenance	Electrical-dominant	5/2/4	40

Note: S/O/D denotes severity, occurrence, and detection. These final consensus values were used in all baseline FRPN calculations.

S3. TFN definitions for linguistic weights

Table S3. Triangular fuzzy number definitions for linguistic weights.

Linguistic weight	TFN	Centroid calculation	Representative value
Medium (M)	(0.3, 0.5, 0.7)	(0.3 + 0.5 + 0.7)/3	0.500
High (H)	(0.5, 0.7, 0.9)	(0.5 + 0.7 + 0.9)/3	0.700
Very high (VH)	(0.7, 0.9, 1.0)	(0.7 + 0.9 + 1.0)/3	0.867

Note: The centroid representative value was used only for normalization of hazard-type-specific S/O/D weights. The integer S/O/D scores were converted using the separate 1-5 TFN scale shown in the main manuscript.

S4. Derivation of hazard-type-specific normalized weights

Table S4. Derivation of hazard-type-specific normalized S/O/D weights and rationale.

Hazard type	Linguistic weights (S/O/D)	Representative values (S/O/D)	Normalization	Normalized weights (wS, wO, wD)	Rationale	Applied steps
Chemical-dominant	H/H/M	0.700, 0.700, 0.500	Sum = 1.900; weights = 0.700/1.900, 0.700/1.900, 0.500/1.900	(0.368, 0.368, 0.263)	Solvent toxicity and vapor exposure can propagate across multiple steps and occur repeatedly during routine handling; therefore, severity and occurrence were emphasized.	1, 2, 4, 11, 12
Electrical-dominant	VH/M/H	0.867, 0.500, 0.700	Sum = 2.067; weights = 0.867/2.067, 0.500/2.067, 0.700/2.067	(0.419, 0.242, 0.339)	High-voltage contact and residual charge can lead to severe consequences, while interlocks, grounding checks, and discharge verification strongly affect detectability.	5, 8, 9, 10, 14
Fire/static-dominant	VH/M/M	0.867, 0.500, 0.500	Sum = 1.867; weights = 0.867/1.867, 0.500/1.867, 0.500/1.867	(0.464, 0.268, 0.268)	Ignition of flammable vapor or static discharge can rapidly escalate, so severity was emphasized.	3
Combined-dominant	VH/H/H	0.867, 0.700, 0.700	Sum = 2.267; weights = 0.867/2.267, 0.700/2.267, 0.700/2.267	(0.383, 0.309, 0.309)	Chemical exposure and electrical ignition sources may become active simultaneously; therefore, severity, occurrence, and detection were all treated as high-priority dimensions.	6, 7, 13

Note: Weights are shown to three decimal places in the manuscript for readability. Internal calculations may use the unrounded values derived from the centroid representative values.

S5. Worked FRPN calculation and baseline calculation sheet

Table S5. Worked FRPN calculation example for Response to Abnormalities (Step 9).

Calculation item	Value
Failure mode	Response to Abnormalities: clog removal without shutdown and discharge confirmation
Consensus S/O/D	5/3/4
Hazard type and weights	Electrical-dominant; wS = 0.4193548, wO = 0.2419355, wD = 0.3387097
TFNs for S/O/D	S = 5 -> (4, 5, 5); O = 3 -> (2, 3, 4); D = 4 -> (3, 4, 5)
Lower vertex L	4^0.4193548 x 2^0.2419355 x 3^0.3387097 = 3.068
Modal vertex M	5^0.4193548 x 3^0.2419355 x 4^0.3387097 = 4.097
Upper vertex U	5^0.4193548 x 4^0.2419355 x 5^0.3387097 = 4.737
Defuzzified value	(3.068 + 4.097 + 4.737)/3 = 3.968
FRPN conversion	3.968 x 20 = 79.35

Note: FRPN was converted to a 100-point scale by multiplying the defuzzified representative value by 20.

Table S5 continued. Baseline FRPN calculation sheet for all representative failure modes.

No.	Process step	Failure mode	Hazard type	S/O/D	RPN	FRPN TFN (L, M, U)	Defuzzified	FRPN
1	Material Review/Approval	Failure to review SDS or omission of additives	Chemical-dominant	5/3/4	60	(2.873, 3.906, 4.605)	3.795	75.89
2	Solution Preparation	Preparation outside the hood or prolonged open preparation	Chemical-dominant	5/3/3	45	(2.582, 3.621, 4.343)	3.515	70.30
3	Solvent Transfer	Transfer of flammable solvent without bonding/grounding	Fire/static-dominant	5/2/4	40	(2.554, 3.685, 4.361)	3.533	70.67
4	Syringe Filling/Installation	Leakage or splashing during syringe/needle connection	Chemical-dominant	4/3/2	24	(1.935, 2.998, 4.026)	2.987	59.73
5	Equipment Setup	Poor collector grounding, exposed high-voltage parts, absence of interlocks	Electrical-dominant	5/3/4	60	(3.068, 4.097, 4.737)	3.968	79.35
6	Pre-Start Inspection	Start-up without confirming exhaust, door status, or distance	Combined-dominant	5/3/4	60	(2.955, 3.986, 4.667)	3.869	77.39
7	Voltage Application/Start-Up	Dripping/arc generation due to excessive voltage, excessive flow rate, or improper distance	Combined-dominant	5/3/3	45	(2.608, 3.648, 4.357)	3.538	70.76
8	Normal Operation	Direct adjustment of the needle/collector during energization	Electrical-dominant	5/2/4	40	(2.595, 3.714, 4.419)	3.576	71.52
9	Response to Abnormalities	Clog removal without shutdown and discharge confirmation	Electrical-dominant	5/3/4	60	(3.068, 4.097, 4.737)	3.968	79.35
10	Shutdown/Discharge	Contact under residual-charge condition after power-off	Electrical-dominant	5/2/4	40	(2.595, 3.714, 4.419)	3.576	71.52
11	Product Collection	Collection of a wet fiber mat on an open bench	Chemical-dominant	4/2/4	32	(2.001, 3.099, 4.142)	3.081	61.62
12	Post-Treatment	Immediate heat treatment of samples with residual solvent	Chemical-dominant	4/2/4	32	(2.001, 3.099, 4.142)	3.081	61.62
13	Waste Disposal/Cleaning	Open waste container and unattended contaminated materials	Combined-dominant	5/2/4	40	(2.385, 3.517, 4.270)	3.391	67.81
14	Maintenance	Opening the power supply or using damaged cables while power remains connected	Electrical-dominant	5/2/4	40	(2.595, 3.714, 4.419)	3.576	71.52

Note: The values in this table match the baseline FRPN ranking reported in the main manuscript. Small last-digit differences can occur if rounded normalized weights are used instead of internal unrounded values.

S6. Post-control recalculation sheet

Table S6. Post-control recalculation sheet for prioritized failure modes.

Process step	Hazard type	Pre-control S/O/D	Pre-control FRPN	Safety-barrier package	Main dimension changed	Post-control S/O/D	Post-control FRPN	Reduction (%)
Response to Abnormalities (9)	Electrical-dominant	5/3/4	79.35	De-energize-discharge-verify procedure; troubleshooting SOP; access control	O and D	5/1/1	44.60	43.79
Equipment Setup (5)	Electrical-dominant	5/3/4	79.35	Grounded enclosure; door interlock; grounding continuity check	O and D	5/1/1	44.60	43.79
Pre-Start Inspection (6)	Combined-dominant	5/3/4	77.39	Pre-start checklist; exhaust/door confirmation; validated operating window	O and D	5/1/2	48.06	37.90
Material Review/Approval (1)	Chemical-dominant	5/3/4	75.89	SDS review gate; composition re-approval; change review	O and D	5/1/2	46.38	38.89
Solvent Transfer (3)	Fire/static-dominant	5/2/4	70.67	Bonding/grounding; sealed transfer; transfer checklist	O and D	5/1/2	52.38	25.88

Note: Severity was retained in these scenarios because the potential maximum consequence was not assumed to change. Occurrence and detection were adjusted only when the barrier package could plausibly reduce exposure opportunity, prevent unsafe intervention, or improve pre-accident detection/verification.

S7. Limited one-at-a-time sensitivity analysis

For the high-priority RPN 60 group, each normalized S/O/D weight was varied by +/-10% one at a time and then re-normalized to sum to one. The same integer S/O/D vector and TFN definitions were retained. This analysis is a local sensitivity check and is not a full robustness assessment of all possible TFN widths or defuzzification schemes.

Table S7. Summary of one-at-a-time sensitivity analysis for the high-priority RPN 60 group.

Failure mode	Hazard type	S/O/D	Baseline weights	Baseline FRPN	FRPN range under +/-10% OAT	Ranking interpretation
Response to Abnormalities (9)	Electrical-dominant	5/3/4	(0.419, 0.242, 0.339)	79.35	78.80-79.91	Remained in joint highest group
Equipment Setup (5)	Electrical-dominant	5/3/4	(0.419, 0.242, 0.339)	79.35	78.80-79.91	Remained in joint highest group
Pre-Start Inspection (6)	Combined-dominant	5/3/4	(0.383, 0.309, 0.309)	77.39	76.79-78.03	Remained below electrical-dominant tied group
Material Review/Approval (1)	Chemical-dominant	5/3/4	(0.368, 0.368, 0.263)	75.89	75.25-76.59	Remained below Pre-Start Inspection

Table S7 continued. Detailed one-at-a-time perturbation results.

Failure mode/group	Perturbation	Re-normalized weights (wS, wO, wD)	FRPN
Electrical-dominant group (Steps 5 and 9)	S -10%	(0.394, 0.253, 0.354)	78.80
Electrical-dominant group (Steps 5 and 9)	S +10%	(0.443, 0.232, 0.325)	79.86
Electrical-dominant group (Steps 5 and 9)	O -10%	(0.430, 0.223, 0.347)	79.91
Electrical-dominant group (Steps 5 and 9)	O +10%	(0.409, 0.260, 0.331)	78.82
Electrical-dominant group (Steps 5 and 9)	D -10%	(0.434, 0.250, 0.316)	79.33
Electrical-dominant group (Steps 5 and 9)	D +10%	(0.406, 0.234, 0.360)	79.37
Pre-Start Inspection (Step 6)	S -10%	(0.358, 0.321, 0.321)	76.83
Pre-Start Inspection (Step 6)	S +10%	(0.405, 0.297, 0.297)	77.91
Pre-Start Inspection (Step 6)	O -10%	(0.395, 0.287, 0.319)	78.03
Pre-Start Inspection (Step 6)	O +10%	(0.371, 0.330, 0.300)	76.79
Pre-Start Inspection (Step 6)	D -10%	(0.395, 0.319, 0.287)	77.30
Pre-Start Inspection (Step 6)	D +10%	(0.371, 0.300, 0.330)	77.46
Material Review/Approval (Step 1)	S -10%	(0.344, 0.383, 0.273)	75.31
Material Review/Approval (Step 1)	S +10%	(0.391, 0.355, 0.254)	76.44
Material Review/Approval (Step 1)	O -10%	(0.383, 0.344, 0.273)	76.59
Material Review/Approval (Step 1)	O +10%	(0.355, 0.391, 0.254)	75.25
Material Review/Approval (Step 1)	D -10%	(0.378, 0.378, 0.243)	75.79
Material Review/Approval (Step 1)	D +10%	(0.359, 0.359, 0.282)	76.00

Note: The electrical-dominant group contains two identical baseline input vectors: Equipment Setup and Response to Abnormalities. Therefore, their FRPN remains tied under the same weight perturbation.

End of Supplementary Materials.
